# Application of X-Ray Powder Diffraction for Analysis of Selected Dietary Supplements Containing Magnesium and Calcium

**DOI:** 10.3389/fchem.2020.00672

**Published:** 2020-09-30

**Authors:** Izabela Jendrzejewska

**Affiliations:** Institute of Chemistry, Faculty of Science and Technology, University of Silesia, Katowice, Poland

**Keywords:** dietary supplements analysis, counterfeit pharmaceutics, X-ray study, Bragg's low, diffraction data, X-ray phase analysis

## Abstract

It is estimated that ~50% of medications and dietary supplements offered in the Internet are counterfeit. X-ray diffraction is one of the techniques which may be successfully applied to identify various chemical compounds in polycrystalline mixtures such as dietary supplements, but also medications, narcotics or designer drugs. X-ray diffraction enables the understanding of compositions of such mixtures. For the tests, 22 dietary supplements which should contain magnesium and calcium compounds, available in pharmacies, groceries, Internet shops, as well as in shops for sportspersons, were selected. Identification of crystalline substances present in the tested sample consists in determination of inter-planar distances *d*_*hkl*_ of investigated substances and determination of intensity of the obtained diffraction lines, and then in comparing them with values contained in diffraction databases. In this study, the ICDD-PDF2 database was used. The most important criterion in qualitative analysis, confirming the presence of a given phase, is the conformity of positions of diffraction lines in the recorded diffraction image with those in the reference image. Reflection shifts for the individual *2*θ angles compared with the data from the database should not exceed 0.2°. In most cases, X-ray analysis of the investigated dietary supplements proved the presence of magnesium and calcium compounds declared by the manufacturer, as well as allowing the identification of auxiliary substances present in the tested products. In the case of two magnesium-containing dietary supplements, the magnesium compounds declared by the manufacturer were not found. Our studies confirmed the effectiveness of X-ray structural analysis and proved the possibility of distinguishing counterfeit preparations from authentic products, as well as to use this method for the quality control of such pharmaceutical preparations.

## Introduction

In recent years, there has been an increasing number of fatal cases resulting from taking counterfeit medical and therapeutical products, and dietary supplements. WHO and FDA experts estimate that counterfeit products may constitute ~10% of the global medicinal drug market. It is thought that the following groups are the largest among counterfeit drugs: antibiotics (28%); hormones (including steroid hormones, 18%); anti-asthmatics and anti-allergics (8%); antimalarials (7%); analgesics and antipyretics (6%); other medications (14 therapeutical classes, 33%) (Maurin et al., 2007; Singh et al., 2009; Venhuis et al., 2011). Also counterfeiters are also interested in popular medicines, such as aspirin. In 2013, 1.2 million aspirin tablets were confiscated in France, and it was a product that did not contain the active substance at all. In the USA, three batches of product containing only water, which were to replace a good, effective, and commonly used oncological drug, were confiscated (World Health Organisation, 2006).

Apart from these, dietary supplements are also massively forged. Because of the fact that they are classified as foodstuffs, their common availability and an increase in the interest for this type of product may be observed at present. More than 10,000 dietary supplements are available on the Polish market. These products contain more than 500 components in total. Use of many of them has no factual substantiation. The majority of plant products, nutrients and dietary supplements is not tested for the quality of the components used. Dietary supplements containing structural analogs of and chemical compounds very similar to those comprised in medicinal drugs, are particularly dangerous. They have much stronger undesirable effects usually and even cause death (World Health Organisation, 2018). That is why it is important to control their chemical composition, using the available test methods (Stypułkowska et al., 2011).

For the study, popular and frequently purchased dietary supplements containing magnesium and calcium were chosen. The goal of the paper consists in the identification of calcium and magnesium compounds declared by manufacturers as components of given supplements, as well as an attempt of determination whether the product is authentic or not.

## Materials and Methods

### Materials

Twenty-two dietary supplements containing calcium and magnesium were purchased in pharmacies, shops, filling stations, and via the Internet, and then subjected to tests using X-ray radiation. All analyzed products are gathered in Table 1. In the table, the data reported by manufacturers (magnesium and calcium contents), and the form of the chemical compound are taken into account.

**Table 1:** Analyzed dietary supplements.

**No.**	**Product name (*manufacturer*)**	**Magnesium content in 1 tablet/sachet [mg]**	**Form of magnesium**
**Analyzed magnesium-containing dietary supplements**			
1.	Falvit *Bausch Health*	112.5	Magnesium oxide
2.	Vitalsss Plus Multivitamin *Natur Product Pharma*	57.0	Magnesium carbonate
3.	Vitalsss Plus Magnez *Natur Product Pharma*	200.0	Magnesium carbonate
4.	Asparoc Apteo *Synoptis Pharma*	17.0	Magnesium carbonate
5.	Vitalsss Plus Magnez + Witamina B_6_ *Natur Product Pharma*	187.5	Magnesium oxide
6.	Magnez OTX *OTXcare*	60.0	Magnesium carbonate
7.	Magnez B_6_ skurcz *INV Poland*	100.0	Magnesium citrate
8.	Dr. Max^+^ Magnez + VitB_6_ *ARENAPHARMA SP. Z O.O*.	60.0	Magnesium lactate Magnesium oxide
9.	Mex Muscle Excellence *MEX Nutrition*	150.0	Magnesium citrate
10.	Thermo Pump *Power Sports Polska*	100.0	Magnesium citrate
11.	7 Nutrition Bomb Pre-workout *TRICEPS Polska*	30.0	Magnesium citrate
12.	Magnesium *KFD Nutrition*	125.0	Magnesium citrate
**Analyzed calcium-containing dietary supplements**			
13.	Calcium plusssz *Polski Lek*	300	Calcium carbonate
14.	Molekin Osteo *NATUR PRODUKT PHARMA*	300	Algae Calcium (*Lithothamnium* sp.) Calcium carbonate
15	Calcium in foil ZDROVIT *NATUR PRODUKT PHARMA*	300	Calcium carbonate
16.	Calcium in foil + vit.C ZDROVIT *NATUR PRODUKT PHARMA*	300	Calcium carbonate
17.	Calcium 500 D *POLFA Łódz*	500	Calcium lactogluconate
18.	Calcium Alergo Plus *POLFA Łódz*	300	Calcium lactate Calcium carbonate
19.	Kalcikinon *VALENTIS*	300	Calcium carbonate
20.	Calcium 400 mg + witamina D3 *VITALIS*	300	Calcium carbonate
21.	Vitrum osteo *Takeda Pharma*	500	Calcium carbonate
22.	Calcium GLUCONICUM *Farmapol*	45	Calcium gluconate

The table does not include auxiliary substances such as starch, talc, magnesium stearate, citric acid, etc., because of the fact that the main goal of the paper is the identification of calcium and magnesium compounds as “active substances” in the analyzed dietary supplements. On the other hand, strong diffraction lines, originating from auxiliary substances, such as ascorbic acid, citric acid, magnesium stearate, alanine, starch, are marked in the diffraction patterns. It pertains particularly to multivitamin preparations, in which the intensity of diffraction lines originating from magnesium and calcium compounds is lower than that of the strongest lines present in the diffraction pattern.

### Methods

#### Diffractometric Method

X-ray radiation has the ability to diffract, or dissipate off the rays on atoms of crystals. A reflection of a beam of parallel rays occurs on a series of parallel lattice planes (*hkl*). The radiation will be amplified when the angle of incidence (θ) is equal to the angle of reflection (θ). To amplify the reflected radiation, the path difference (Δs) must be equal to a total multiple of the wavelength (nλ), as only then, the wave are in phase. The scheme of X-ray diffraction is shown in Epp (2016).

The amplification condition will be met if:

(1) $$\begin{array}{l} n\lambda =2d_{hkl}sin\theta \end{array}$$

where: *n*—reflection order, λ—wavelength, *d*_*hkl*_—interplanary distance, θ—angle of reflection.

**Figure 1:**
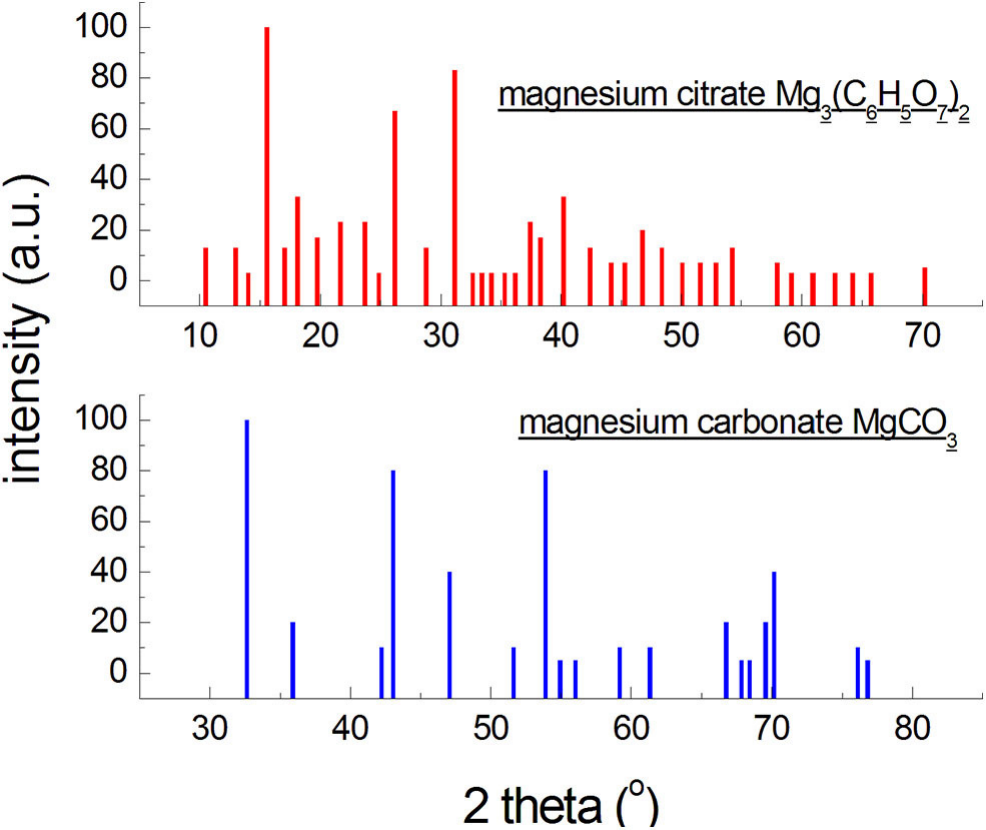
Diffraction patterns generated based on the data from the ICDD database for an organic and an inorganic magnesium salt.

The Equation (1), namely the Bragg-Wulff equation, describes the geometrical condition for X-ray diffraction (XRD) on lattice planes having interplanary distances of *d*_*hkl*_. An important advantage of the method consists in the fact that not only the θ angle, at which the X-rays are being reflected is measured, but also the intensities of the observed diffraction pattern lines. The diffraction pattern is obtained in the form of a plot of the intensity (count number) *vs*. the *2*θ (deflection angle) (Figure 1).

#### X-Ray Structural Analysis

X-ray phase analysis plays an important role in the analysis of almost all solids, including medications and medicinal preparations. As a non-destructive method, it is used for: (a) identification of unknown compounds, (b) investigation of polymorphism, solvation and salt form identification, (c) determination of physico-chemical properties, (d) detection of impurities and anomalies in medications (Thatcher and Briner, 1986; Stephenson, 2005; Chauhan and Chauhan, 2014; Das et al., 2014). X-ray phase analysis enables both qualitative and quantitative analyses of polycrystalline substances. Each crystalline substance has a characteristic X-ray diffraction pattern with specific locations of diffraction lines and their intensities. Such polycrystalline diffraction patterns are so complex that it is not possible to have identical diffraction images for various substances. Therefore, diffraction patterns may play the role of “fingerprints” in the identification of substances. A diffraction pattern may be considered as a set of interplanary distances *d*_*hkl*_ and line intensities corresponding to them. It is important that every phase included in the composition of the mixture is independent in the process of creation of the diffraction pattern, which is a sum of diffraction patterns of the co-existent phases. To carry out the qualitative phase analysis, one should compare the obtained diffraction pattern (*2*θ angles, *d*_*hkl*_ interplanary distances, and diffraction line intensities) with a corresponding standard, found in a proper database. A diffraction line shift of <0.2° is a normal phenomenon while analyzing a polycrystalline substance. It is connected with a random arrangement of grains in a polycrystalline sample. On the other hand, shifts larger than 0.2° at a given diffraction angle *2*θ indicate a different crystal structure. Counterfeit pharmaceuticals contain various types of auxiliary substances (excipients) with different crystal structures than those of substance present in authentic products (Kugler, 2003; Rendle, 2003). Based on this assumption, a product for which the obtained diffraction lines are shifted by more than 0.2°, should be considered suspect (USP Pharmacopeial Convention, 2011; DeWitt, 2015). This rule is published in the general chapter of the <941> ascertaining that if the shifts of diffraction lines in an XRD image of the tested products are larger than 0.2° for a given *2*θ diffraction angle while compared to the XRD image for an authentic product, these products meet the counterfeit criteria (DeWitt, 2015). Also, counterfeit drugs and supplements may be distinguished from authentic ones by studying their general XRD images. Additional lines, lack of lines, as well as line shifts will be observed in diffraction patterns of counterfeit products. This method enables the application of X-ray phase analysis as a technique for distinguishing counterfeit pharmaceuticals from authentic ones in legal chemistry.

The obtained diffraction pattern contains information on the angle of reflection θ and wavelength λ, so, using the Bragg Equation (1), the interplanary distance *d*_*hkl*_ may be calculated (Bojarski and Łagiewka, 1988). The obtained values of the interplanary distances *d*_*hkl*_ characterize a given phase and are independent from the radiation type, while the line intensities are closely related to it. Due to this method, identification of components of crystalline phases containing the tested material is possible, and simultaneously, qualitative analysis of the sample takes place.

There is a dependence between the intensity $J_{hkl}^{j,0}$ of any *j* phase reflection, having the mass absorption coefficient $\mu_{j}^{*}$, and the intensity of the same reflection, $J_{hkl}^{j}$, in the case when the phase *j* with a mass share *m*_*j*_ is present in a polyphasic mixture with a mass coefficient μ^*^, shown by the following formula:

(2) $$\begin{array}{l} J_{hkl}^{j}=J_{hkl}^{j,0}\frac{\mu_{J}^{*}}{\mu^{*}}m_{j} \end{array}$$

As can be seen from the formula (2), the intensity of a recorded reflection of a given phase depends on its amount in the mixture *m*_*j*_ and the $J_{hkl}^{j,0}$ value, or its crystal structure (Bojarski and Łagiewka, 1988).

Identification of phases in polyphasic mixtures (and dietary supplements should be considered such) depends on crystal structure of a given phase, characters of the co-existing phases, and instrumental factors. X-ray photographs of crystalline phases having a high symmetry (regular, tetragonal, hexagonal systems) contain a relatively low number of diffraction reflections, but of high intensities. It allows for identifying them even at their contents below 1%. It is assumed that the X-ray detection limit is in the range of 0.1–1% by wt. per phase, while the limit of detection (LOD) is assumed as ~1% (Bojarski and Łagiewka, 1988)^1^.

On the other hand, X-ray photographs of low-symmetry phases (triclinic, monoclinic systems) contain large numbers of reflections, but of low intensities, leading to worse detection limits. Phases composed of atoms of elements having high atomic numbers will exhibit higher intensities of reflections than those composed of light elements (Figure 1). It is a consequence of atomic dissipation factor, increasing with the increase in the atomic number of the element. Thus, the X-ray detection limit will be more favorable for compounds with a high symmetry, and unfavorable for compounds with a low symmetry composed of light elements. It should be noted that more than 2/3 of organic compounds crystallize in low-symmetry systems, and the strongest diffraction lines are observed at small angles (Figure 1). The content of a given crystalline phase may be lower than this limit, so it will not be identified, but it does not mean it is absent. The detection limit is affected also by size of the crystallites and perfection of the crystal lattice. Defected lattices and crystallite sizes below 0.1 μm cause broadening and weakening of intensities of the reflections, hindering identification. The change in the limit of detection of a crystalline phase, depending on the type of the mixture, wherein this phase is present, consists in a change in the ration between the absorption coefficient of the phase and the mixture as a whole, $\mu_{j}^{*}/\mu^{*}$, and in a superposition of reflections of the concomitant phases. The detection limit of phase *j*, having an absorption coefficient $\mu_{j}^{*}$, will be more favorable in a mixture with a low absorption coefficient than in a mixture with a high value of absorption coefficient (formula 2) (Bojarski and Łagiewka, 1988).

Quantitative analysis is based on the diffraction line intensity expressed as formula (3):

(3) $$\begin{array}{l} J_{hkl}=C{\left|F_{hkl}\right|}^{2}\cdot LP\cdot p\cdot A \end{array}$$

where: C—constant, F_hkl_–structural factor, LP—Lorentz, and polarization factor, p—plane multiplicity factor, A—absorption factor.

Depending on the number of phases in the mixture and their relationships, several typical methods of quantitative analysis are distinguished: (i) direct comparison of reflection intensities, (ii) internal standard method, (iii) external standard method, (iv) Chung method. It is important that reflections having adequate intensities are chosen. These should be the strongest, well separated reflections, located in a small angle range. Precision of quantitative X-ray analysis depends on many factors, therefore this method is affected by errors related to the structure of the phase being determined and the preparation of the sample. Precision of this method ranges from tenths of per cent to several per cent, depending on the analyzed mixture (Bojarski and Łagiewka, 1988).

#### Sample Analysis of Dietary Supplements

Our studies were focused on a qualitative analysis of selected dietary supplements containing calcium and magnesium compounds. Samples of dietary supplements were very finely ground in an agate mortar, until a homogeneous fine powder was obtained. The tests were carried out using a PW1050 polycrystalline diffractometer with a PW1729 generator from Philips. Bragg-Brentano focusing of diffractive radiation was applied. The total duration of the analysis of each supplement amounted to 48 h, the angular range of the goniometer: 5°÷135°, CuKα1 radiation (λ = 1.54056 Å), filter—Ni. During the experiment, a full scan in the angle range of 5°–120° was carried out, with an angular step of 0.05°, and the scanning time was 0.1 s. The next measurement was configured so that the angle range matched to the given preparation subjected to the analysis. When the tested sample did not exhibit any peaks above 80°, the second measurement was recorded in the 2θ angle range of 5°÷80° or 10°÷80°. Parameters of the second measurement were as follows: 0.02° angular step and 0.02 s scanning time, affecting the quality of the diffraction pattern distinctly. The measurement was carried out twice or thrice to eliminate all errors.

## Results and Discussion

Identification of calcium and magnesium compounds contained in the tested supplements was performed based on the data from the ICDD PDF−2 database (Release 2008, Table A1). For each tested substance, qualitative phase analysis was carried out. It consisted in a comparison of the experimental diffraction data such as *2*θ diffraction angles, *d*_*hkl*_ interplanary distances, and relative intensities, with the data from the ICDD database. Values of *d*_*hkl*_ interplanary distances were calculated based on the Bragg-Wulff equation. The results gathered in tables and shown in the figures are grouped according to the magnesium or calcium compound contained in the tested dietary supplement.

### Phase Analysis of Dietary Supplements Containing Magnesium

**Figure 2:**
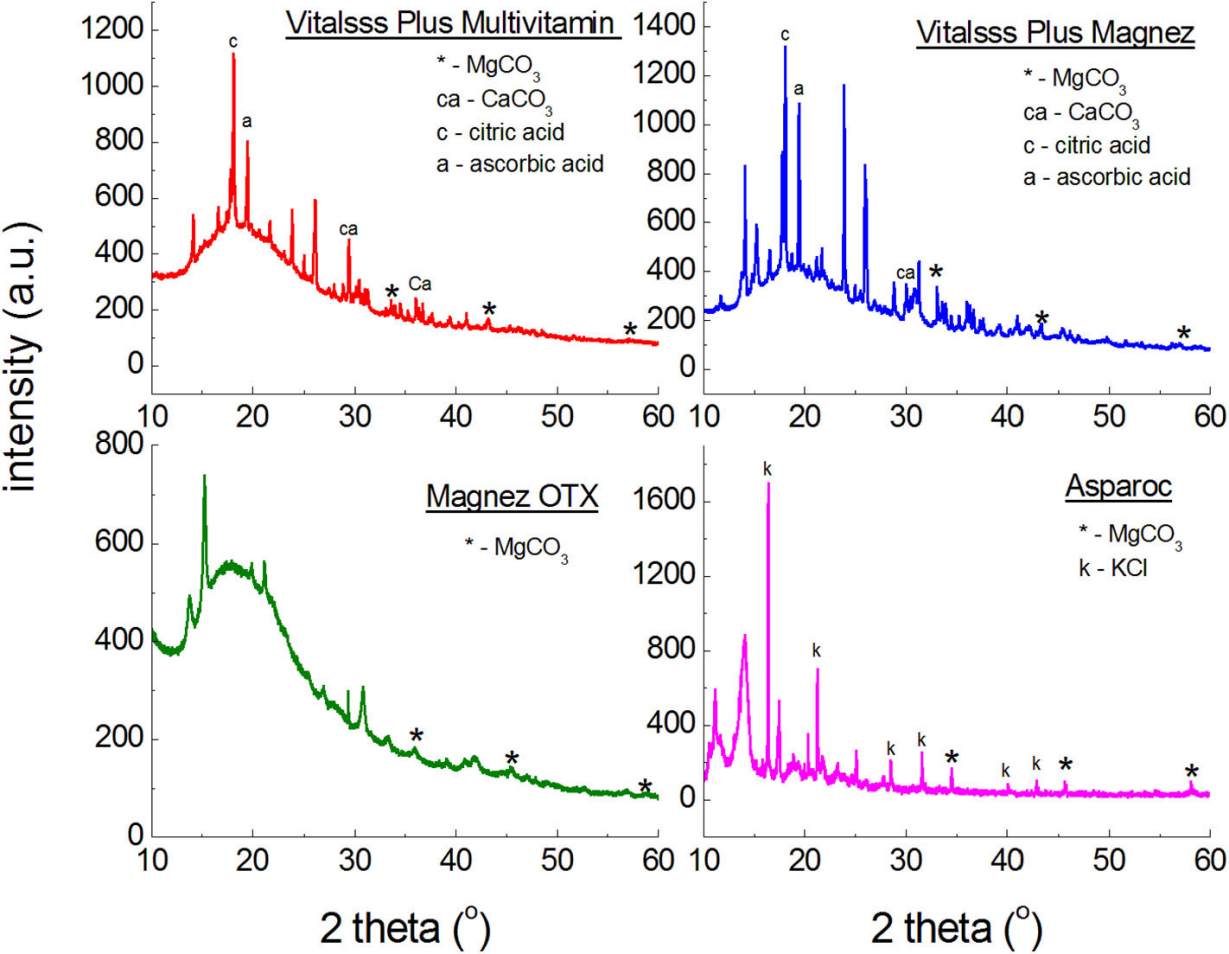
X-ray powder diffraction spectra of dietary supplements containing magnesium carbonate (MgCO_3_).

Figure 2 illustrates polycrystalline diffraction patterns for the following dietary supplements: *Asparoc APTEO Vitalsss Plus Multiwitamina, Vitalsss Plus Mg* + *K*, and *Magnez OTX*. In all diffraction patterns, diffraction lines characteristic for magnesium carbonate (MgCO_3_) are present. Their intensity is small, confirming the small amount of MgCO_3_ declared by the manufacturer (Table 1). The largest amount of MgCO_3_ is contained in Vitalsss Plus Magnesium (200 mg) and it is evident in the diffraction pattern, where the lines originating from MgCO_3_ have the highest intensity in comparison to the other dietary supplements with MgCO_3_.

The comparison of experimental values of the *2*θ angle with the data from the ICDD database for magnesium carbonate showed that the peak shifts are smaller than 0.2° (Table 2). It proves that the same crystalline form of MgCO_3_, thus the same product, is present in every tested dietary supplement, meaning that the tested preparation are authentic.

**Table 2:** Comparison of experimental data with the data from the ICDD database for the following dietary supplements: *Vitalsss Plus Multiwitamina, Vitalsss Plus Magnez, Magnez OTX, Asparoc* containing magnesium carbonate (MgCO_3_).

**No. of diffraction line**	**2θ (°) exp.**	**2θ (°) ICDD**	**Intensity**	**Δ2θ**	**d_hkl_ (Å) exp.**	**d_hkl_ (Å) ICDD**	**hkl**
***Asparoc APTEO*** **(MgCO**_**3**_ **PDF 01–070–8513)**							
1.	34.4519	34.5963	100	0.1446	2.60	2.59	104
2.	44.7239	44.8843	55	0.1604	2.02	2.02	113
3.	58.0396	57.8903	23	0.1493	1.59	1.61	116
***Vitalsss Plus Multiwitamina, Vitalsss Plus Magnez*** **(MgCO**_**3**_ **PDF 01–070–8513)**							
1.	34.4527	34.5963	100	0.1736	2.60	2.59	104
2.	44.7412	44.8843	55	0.1431	2.02	2.02	113
***Magnez OTX*** **(MgCO**_**3**_ **PDF 01–070–8515)**							
1.	35.8432	35.6711	100	0.1721	2.51	2.51	104
2.	45.6132	45.9207	70	0.3075	1.98	1.97	113
3.	58.7563	58.8903	30	0.1340	1.57	1.57	116

**Figure 3:**
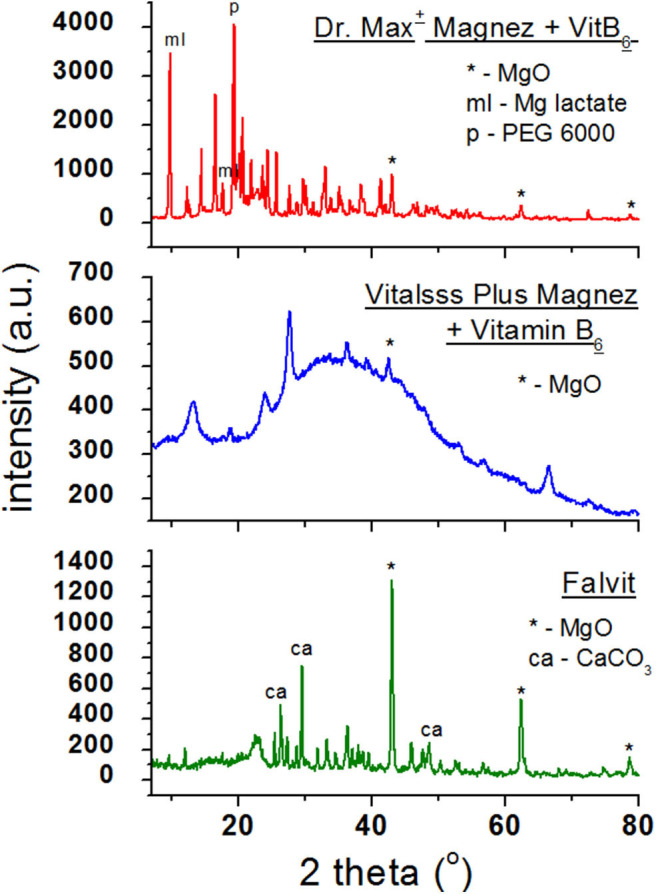
X-ray powder diffraction spectra of dietary supplements containing magnesium oxide (MgO).

Figure 3 presents diffraction patterns of dietary supplements containing magnesium oxide (MgO). In the diffraction patterns of *Falvit* and *Dr. Max*^+^ *Magnez* + *VitB*_6_ (Figure 2), three of the strongest diffraction lines characteristic for magnesium oxide MgO are clearly evident. For *Vitalsss Plus Magnez*, only one line originating from MgO was identified, at the *2*θ corresponding to the strongest line for MgO. Intensity of this line was significantly lower than that of the MgO line in *Falvit* preparation, however, the amount of MgO in *Vitalsss Plus Magnez* is higher in comparison with that in *Falvit* (Table 1). It indicates that the amount of magnesium oxide in the former preparation is lower than the manufacturer declares.

Comparing the experimental *2*θ values and the calculated values of interplanary distances *d*_*hkl*_ gathered in Table 3 with the data from the ICDD database, their very good accordance may be confirmed. The shifts of the reflections for the individual diffraction angles *2*θ compared to the reference data are smaller than 0.2°, confirming the authenticity of the product.

**Table 3:** Comparison of experimental data with the data from the ICDD database for dietary supplements *Falvit* and *Vitalsss Plus Mg* + *vit. B*_6_ containing magnesium oxide (MgO) and *Dr. Max*^+^ *Magnez* + *VitB*_6_ containing also magnesium lactate (C_6_H_10_MgO_6_3H_2_O) apart from magnesium oxide (MgO).

**No. of diffraction line**	**2θ (°) exp.**	**2θ (°) ICDD**	**Intensity**	**Δ2θ**	**d_hkl_ (Å) exp.**	**d_hkl_ (Å) ICDD**	**hkl**
***Falvit* (MgO PDF 00–001–1235)**							
1.	43.0245	43.0368	100	0.0123	2.10	2.10	111
2.	62.3951	62.2582	75	0.1369	1.49	1.49	220
3.	78.9413	79.0768	15	0.1355	1.21	1.21	222
***Vitalsss Plus Mg + vit. B*_6_ (MgO PDF 00–001–1235)**							
1.	42.8321	43.0368	100	0.2047	2.10	2.10	111
***Dr. Max^+^ magnesium + VitB*_6_ (MgO PDF 00–001–1235)**							
1.	42.9623	43.0368	100	0.0763	2.10	2.11	200
2.	62.4159	62.2582	52	0.1577	1.49	1.49	220
3.	78.8806	79.0768	15	0.1962	1.21	1.21	222
***Dr. Max^+^ Magnez + VitB*_6_ (C_6_H_10_MgO_6_3H_2_O PDF 00–001–0061)**							
1.	9.4976	9.3015	100	0.1961	9.30	9.50	–
2.	17.5541	17.3739	80	0.1802	5.05	5.10	–

**Figure 4:**
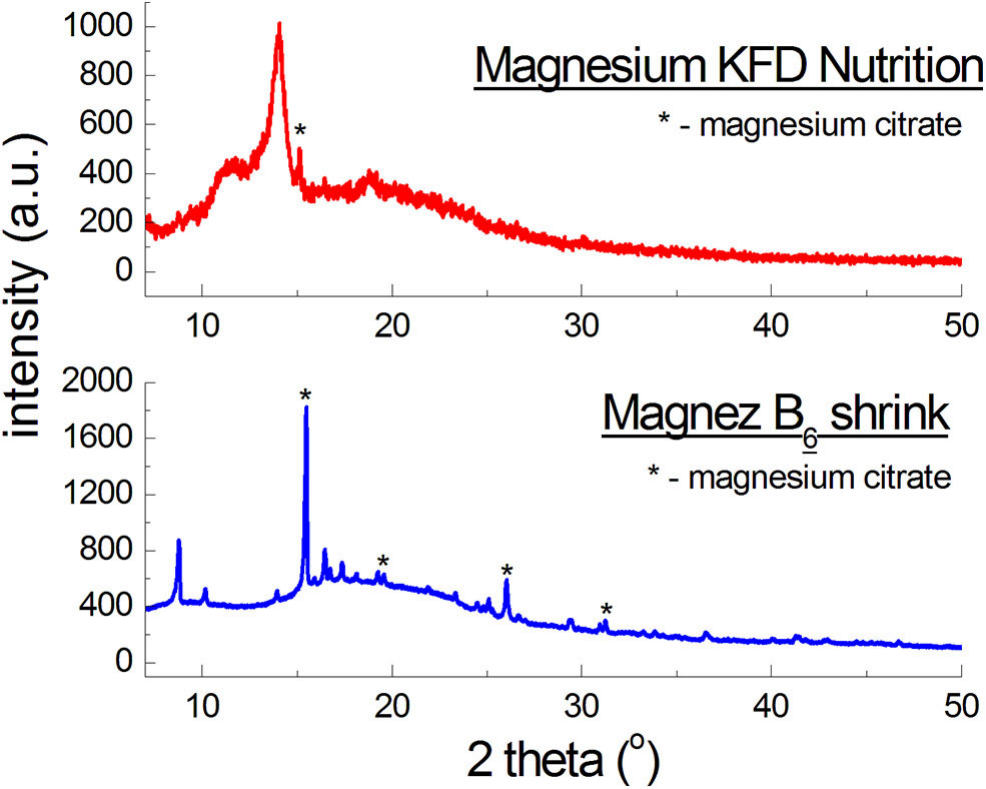
X-ray diffraction pattern for dietary supplements containing magnesium citrate [Mg_3_(C_6_H_5_O_7_)_2_].

Figure 4 presents diffraction patterns of dietary supplements with magnesium citrate. For *Magnez B*_6_ *skurcz*, 4 well-evident diffraction lines were identified, while for *Magnesium KFD Nutrition* only one line is visible for an angle close to the *2*θ value, at which the strongest line from magnesium citrate is observed. The line has a lower intensity than, approximately, the corresponding line for *Magnez B*_6_ *skurcz*, despite the fact that the amount of magnesium citrate is relatively larger in *Magnesium KFD Nutrition* (Table 1). It may indicate a significantly smaller amount of magnesium citrate in *Magnesium KFD Nutrition* than the amount declared by the manufacturer.

The experimental data (*2*θ angle values and the calculated values of *d*_*hkl*_ interplanary distances) for *Magnez B*_6_ *skurcz* are in a good accordance with the data from the ICDD database (Table 4). The Δ*2*θ differences for the individual diffraction angles *2*θ while compared with the ICDD data are smaller than 0.2°, which may prove that the tested preparation is authentic. On the other hand, for *Magnesium KFD Nutrition*, the Δ*2*θ value is higher than 0.2°, possibly indicating irregularities in the composition of this supplement, e.g., a lack of magnesium citrate, an amount lower than that declared by the manufacturer, or presence of another substance giving a diffraction pattern close to that of magnesium citrate.

**Table 4:** Results of analysis of experimental data and data from the ICDD database for the following dietary supplements: *Magnez B*_6_ *skurcz* and *Magnesium KFD Nutrition* and for sportspersons: *Mex Muscle Excellence* and *Thermo Pump* [magnesium citrate Mg_3_(C_6_H_5_O_7_)_2_, PDF 00–001–0186].

**No. of diffraction line**	**2θ (°) exp.**	**2θ (°) ICDD**	**Intensity**	**Δ2θ**	**d_hkl_ (Å) exp.**	**d_hkl_ (Å) ICDD**	**hkl**
***Magnez B*_6_*skurcz***							
1.	15.3818	15.5331	100	0.1513	5.76	5.70	–
2.	18.1052	18.0889	33	0.0163	4.90	4.90	–
3.	26.1338	26.1884	67	0.0546	3.41	3.40	–
4.	31.2150	31.1370	83	0.0780	2.86	2.87	–
***Magnesium KFD Nutrition***							
1.	15.1703	15.5331	100	0.3628	5.83	5.70	–
***Mex Muscle Excellence***							
1.	15.0362	15.5331	100	0.2969	5.89	5.70	–
2.	26.1465	26.1884	67	0.0419	3.40	3.40	–
3.	31.1596	31.1370	83	0.0226	2.87	2.87	–
***Thermo Pump***							
1.	15.2275	15.5331	100	0.3103	5.81	5.70	–
2.	26.4399	26.1884	67	0.2515	3.37	3.40	–
3.	31.0575	31.1370	83	0.0795	2.88	2.87	–

**Figure 5:**
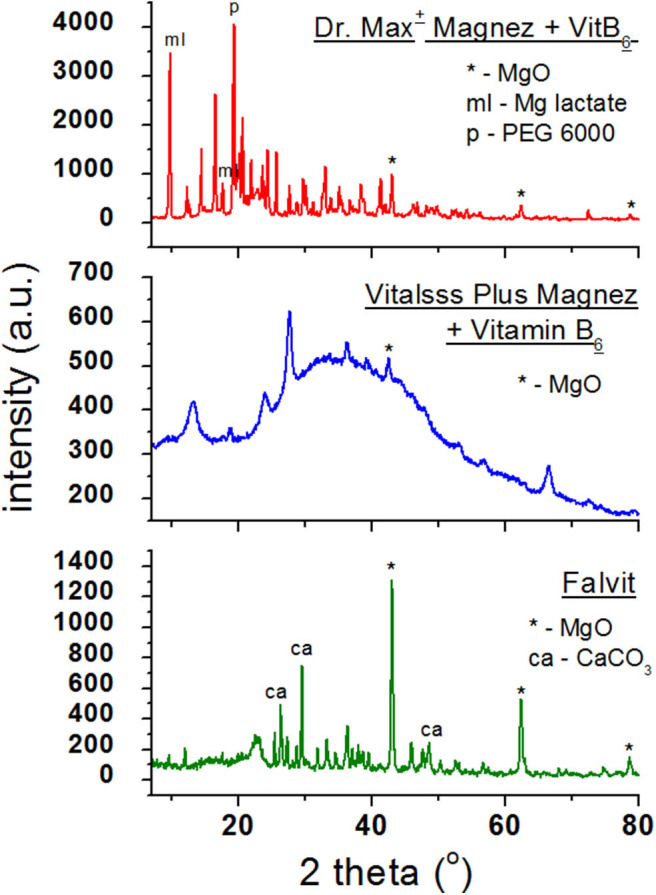
X-ray diffraction pattern for dietary supplements containing magnesium citrate [Mg_3_(C_6_H_5_O_7_)_2_].

In Figure 5, diffraction patterns for multicomponent dietary supplements intended for people engaging in sports are shown. The magnesium-supplementing compound is magnesium citrate. Diffraction lines characteristic for magnesium citrate are present in the diffraction patterns of *Mex Muscle Excellence* and *Thermo Pump*. Comparison of intensities of lines characteristic for magnesium citrate shows a significant decrease in the intensity of the *Thermo Pump* line, despite the relatively high content of this compound (Table 1). It indicates a lower amount of magnesium citrate in *Thermo Pump* than that declared by the manufacturer. Comparing the experimental values of *2*θ angles and the calculated values of interplanary distances *d*_*hkl*_ gathered in Table 4 with the data from the ICDD database, their very good accordance may be confirmed. The shifts of the reflections for the individual *2*θ diffraction angles are smaller than 0.2°, proving that magnesium citrate is present in the tested preparations.

In the case of *7 Nutrition Bomb Pre-workout* dietary supplement for sportspersons, no lines originating from—Mg_3_(C_6_H_5_O_7_)_2_–were found (Figure 5). All visible lines originated from alanine—one of the components of the analyzed product according to its manufacturer. The identified alanine constitutes 25% (5,000 mg) of the total product mass. In this case, the problem of identification of magnesium citrate is probably connected with the composition of the analyzed preparation for sportspersons. Taking into account the information placed on the packaging, the preparation's composition includes more than 20 various substances. According to this description, the total amount of the preparation should contain only 0.15% (30 mg) of magnesium citrate, meaning that the magnesium citrate content may be lower than the roentgenographic detection limit.

### Phase Analysis of Dietary Supplements Containing Calcium

**Figure 6:**
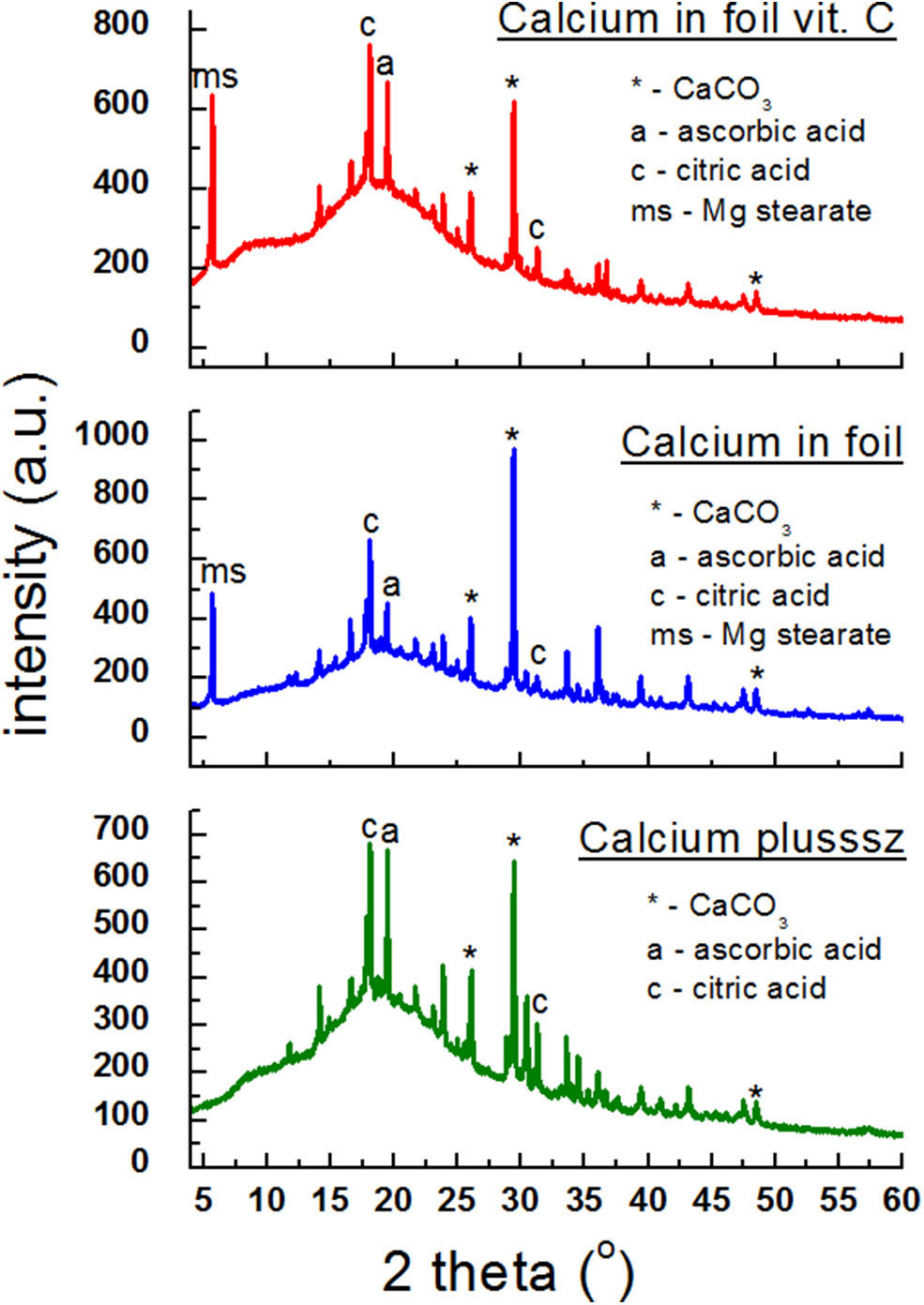
X-ray diffraction pattern for dietary supplements containing calcium carbonate (CaCO_3_) and ascorbic acid.

In Figure 6, diffraction patterns of the following dietary supplements are shown: *Calcium plusssz, Calcium in foil, Calcium in foil* + *vit.C* containing calcium carbonate CaCO_3_ as a compound introducing calcium ions to the human organism, in the presence of ascorbic acid. Ascorbic acid was identified in *Calcium plusssz* and *Calcium in foil*, despite the fact that the manufacturer did not declare this component. Lines of citric acid, responsible for the taste of these supplements, have high intensities. In the diffraction patterns of the analyzed supplements (Figure 6), three of the strongest diffraction lines characteristic for calcium carbonate (CaCO_3_) are clearly visible. The intensities of these lines are similar, confirming the amount of calcium carbonate declared by the manufacturer.

Table 5 presents a comparison of diffraction tests for the following dietary supplements: *Calcium plusssz, Calcium in foil, Calcium in foil* + *vit.C*. Comparison of experimental values of *2*θ angles with the data from the ICDD database for calcium carbonate showed a very good accordance with the standard. The determined shifts Δ*2*θ of the peaks are significantly smaller than 0.2°. It proves that the same crystalline form of CaCO_3_ is present in these supplements, meaning that the tested sample is authentic.

**Table 5:** Comparison of experimental data with the data from the ICDD database for the following dietary supplements: *Calcium plusssz, Calcium in foil, Calcium in foil* + *vit.C, Kalcikinon, Calcium 400 mg* + *vit. D3 VITALIS, Vitrum Osteo* (CaCO_3_ PDF 00–001–0837).

**No. of diffraction line**	**2θ (°) exp.**	**2θ (°) ICDD**	**Intensity**	**Δ2θ**	**d_hkl_ (Å) exp.**	**d_hkl_ (Å) ICDD**	**hkl**
***Calcium plusssz, Calcium in foil, Calcium in foil + vit.C***							
1.	26.0961	26.1884	100	0.0923	3.41	3.40	111
2.	29.3592	29.3554	100	0.0038	3.04	3.04	104
3.	48.4648	48.3749	60	0.0899	1.87	1.88	202
***Kalcikinon, Calcium 400 mg + vit. D3 VITALIS, Vitrum Osteo***							
1.	22.9932	23.0218	8	0.0286	3.86	3.86	012
2.	29.4363	29.3554	100	0.0809	3.03	3.04	104
3.	35.8795	36.0401	20	0.1606	2.50	2.49	110
4.	39.3147	39.4909	24	0.1762	2.29	2.28	113
5.	43.1593	43.2530	10	0.0937	2.09	2.09	202
6.	47.4802	47.3049	32	0.1753	1.91	1.92	024
7.	48.8083	48.6503	24	0.1580	1.86	1.87	116
8.	57.7010	57.5570	16	0.1440	1.60	1.60	122
9.	62.7736	62.7263	12	0.0473	1.48	1.48	119
10.	64.8629	64.6766	5	0.1863	1.43	1.44	125

**Figure 7:**
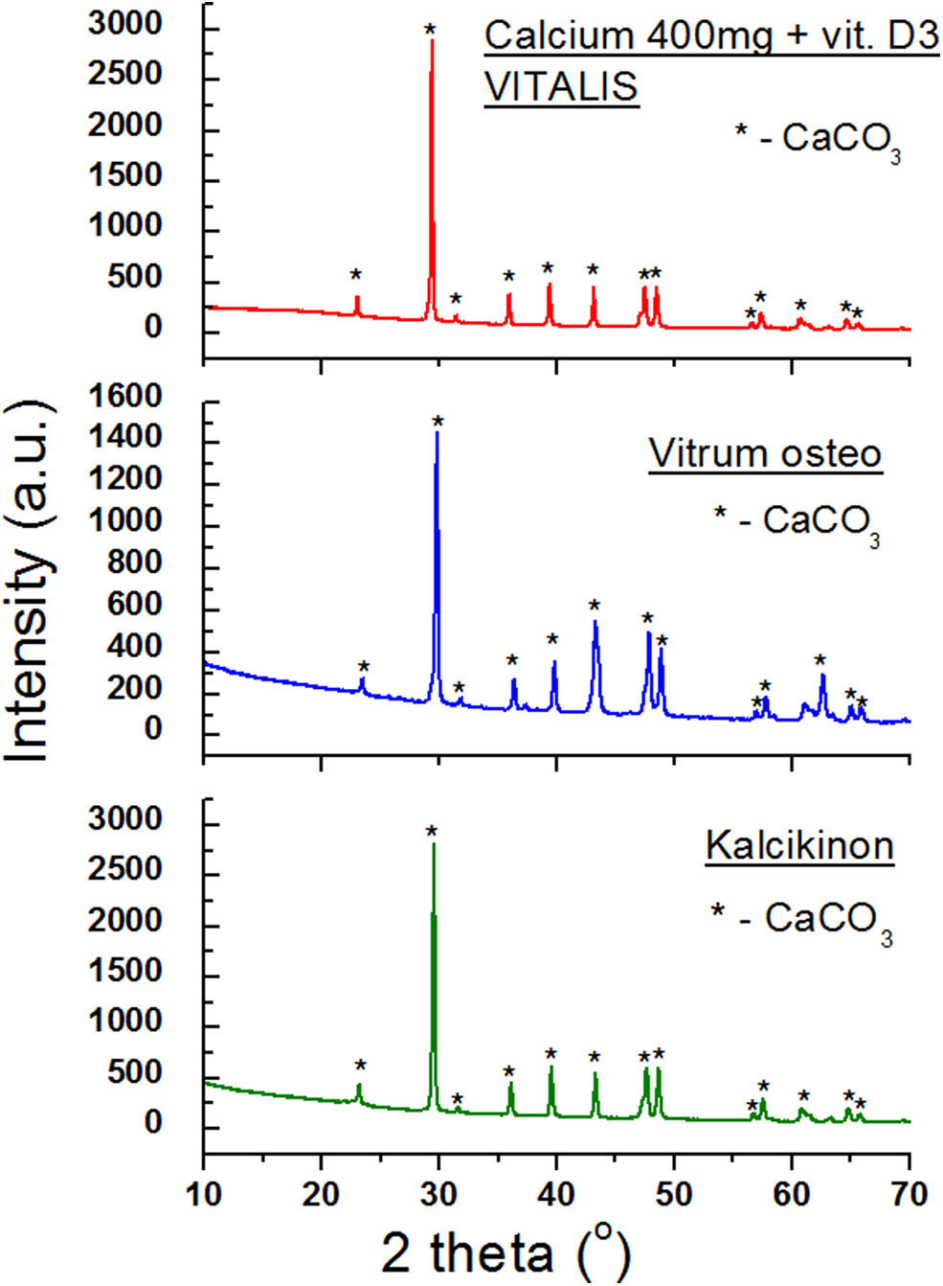
X-ray diffraction patterns for dietary supplements containing calcium carbonate CaCO_3_.

Figure 7 presents diffraction patterns of the next dietary supplements containing CaCO_3_. In the case of these three preparations, calcium carbonate is basically the sole component. Only diffraction lines from CaCO_3_ are present in the diffraction patterns (Figure 7). No lines from auxiliary substances (excipients) were found, indicating that the amounts of these substances are significantly lower than the detection limit or they are not present at all.

Analysis of values of *2*θ angles read from the diffraction patterns is in a very good accordance with the data contained in the database, proving that the same crystalline variety of CaCO_3_ is present in all three products. This good accordance has been confirmed also by the determined Δ*2*θ difference, which does not exceed 0.2° (Table 5).

**Figure 8:**
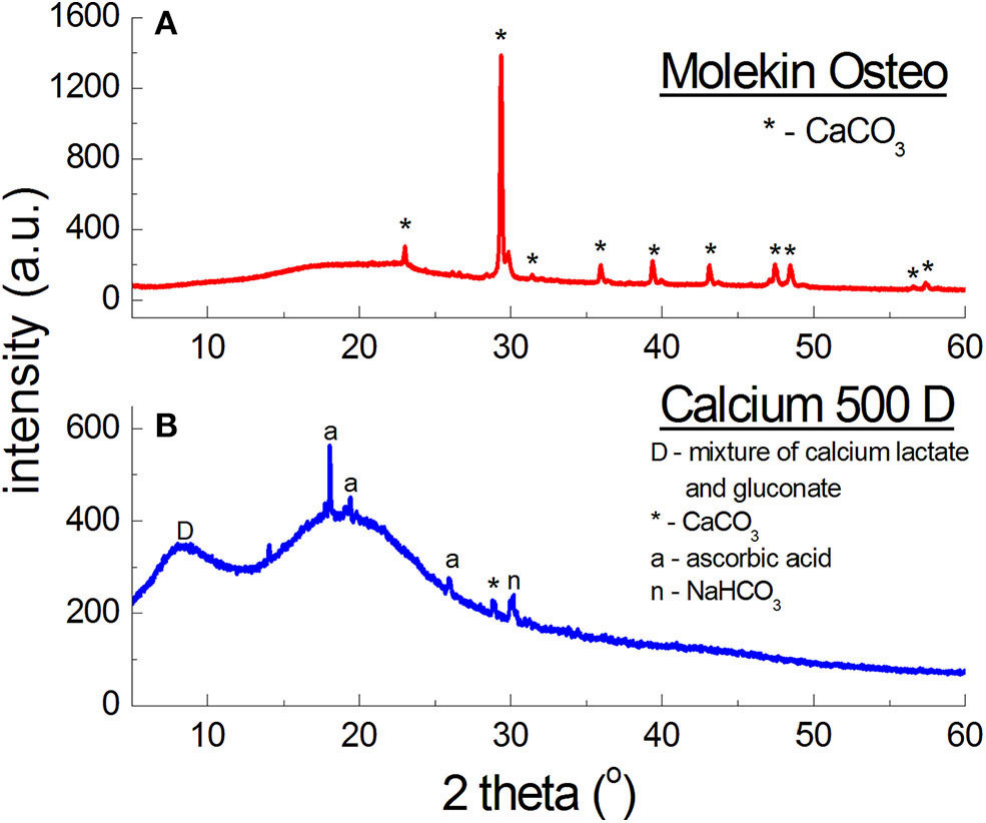
X-ray diffraction pattern for dietary supplements containing organic salts of calcium **(A)** and calcium carbonate CaCO_3_ and a mixture of organic salts of calcium **(B)**.

Figure 8A presents diffraction patterns of *Calcium Alergo Plus* and *Calcium Gluconicum* dietary supplements. According to the manufacturer's information, the active substance of *Calcium Alegro Plus* is constituted by a mixture of an organic and an inorganic calcium salt (calcium lactate and calcium carbonate). CaCO_3_ is the main component of this preparation; 7 diffraction lines of this compound were identified as having intensities concordant with the ICDD database. For calcium lactate, only one diffraction line with a low intensity was found. The highest intensity in the obtained diffraction pattern is exhibited by the line of SiO_2_, being a filler.

Polycrystalline diffraction pattern of *Calcium Gluconicum* consists of many diffraction lines, which are difficult to identify (Figure 8A). These lines may originate from auxiliary substances not listed in the composition of this supplement. The active substance of *Calcium Gluconicum* is calcium gluconate. Diffraction lines of this component were identified in the diffraction pattern. On the other hand, the strongest visible diffraction line originates from starch.

For both supplements, *Calcium Alergo Plus* and *Calcium Gluconicum*, a very good accordance of experimental values of *2*θ angles and the values of *2*θ angles from the ICDD database is evident (Table 6). The determined values of Δ*2*θ are lower than 0.2°, proving the authenticity of the product.

**Table 6:** Comparison of experimental data with the data from the ICDD database for the following dietary supplements: *Calcium Alergo Plus, Calcium Gluconicum, Molekin Osteo*, and *Calcium 500D*.

**No. of diffraction line**	**2θ (°) exp**.	**2θ (°) ICDD**	**Intensity**	**Δ2θ**	**d_hkl_ (Å) exp**.	**d_hkl_ (Å) ICDD**	**hkl**
***Calcium Alergo Plus – calcium lactate* (PDF 00–029–1526)**							
1.	21.9172	21.9830	50	0.0658	4.05	4.04	–
***Calcium Alergo Plus – CaCO*_3_ (PDF 00–001–0837)**							
1.	23.0303	23.0218	8	0.0850	3.86	3.86	012
2.	29.3939	29.3554	100	0.0385	3.04	3.04	104
3.	35.9557	36.0401	20	0.0844	2.50	2.49	110
4.	39.4638	39.4909	24	0.0271	2.28	2.28	113
5.	43.1303	43.2530	10	0.1227	2.10	2.09	202
6.	47.4825	47.3049	32	0.1776	1.91	1.92	024
7.	48.5081	48.6503	24	0.1422	1.86	1.87	116
***Calcium Gluconicum – calcium gluconate* (PDF 00–010–0774)**							
1.	16.1868	16.1607	70	0.0261	5.47	5.48	110
2.	19.4794	19.4072	40	0.0722	4.55	4.57	110
3.	24.4619	24.3661	50	0.0958	3.64	3.65	202
***Molekin Osteo – CaCO*_3_ (PDF 00–001–0837)**							
1.	23.0128	23.0218	8	0.0090	3.86	3.86	012
2.	29.3840	29.3554	100	0.0286	3.04	3.04	104
3.	35.9722	36.0401	20	0.0679	2.49	2.49	110
4.	39.3589	39.4909	24	0.1320	2.29	2.28	113
5.	43.1624	43.2530	10	0.0906	2.09	2.09	202
6.	47.4786	47.3049	32	0.1737	1.91	1.92	024
7.	48.5256	48.6503	24	0.1247	1.87	1.87	116
8.	57.4038	57.5570	16	0.1537	1.60	1.60	122
***Calcium 500D – mixture of calcium lactate and gluconate* (PDF: 00-029-1596, 00-010-0774)**							
1.	8.2372	8.9069	100	–	–	–	
		9.9751	100				–

The diffraction pattern of *Molekin* dietary supplement (Figure 8B) contains lines of only CaCO_3_, however, the manufacturer declares also “calcium from seaweed” (in an unknown form). X-ray phase analysis clearly indicates presence of only calcium carbonate (CaCO_3_), similarly as in the preparations described in Table 6.

On the other hand, in *Calcium 500D* (Figure 8B) the source of calcium ions according to the manufacturer's claims, was constituted by calcium lactate gluconate, which could not be determined unequivocally in phase analysis. it is probable that a mixture of organic calcium salts: gluconate and lactate, form a broad diffraction peak with a maximum *2*θ = 8.7190°, because the strongest line for calcium gluconate occurs at the angle of *2*θ = 9.9751°, and for calcium lactate—at *2*θ = 8.9521°.

The shape of the line, its width, and maximum value depend on the amounts of lactate and gluconate in this mixture. The manufacturer does not report these values. However, lines originating from calcium carbonate were identified in *Calcium 500D*, this compound likely being the main source of calcium ions in this preparation. Apart from the lines of CaCO_3_, peaks originating from citric acid, affecting the taste of the supplement, were identified. The manufacturer claims that 1 sachet contains 500 mg of calcium in the form of 3.875 g of calcium lactate gluconate, and 60 mg of ascorbic acid. X-ray analysis shows that the amount of calcium ions is significantly lower. It is proved by intensities of the lines originating from the mixture of calcium lactate and calcium gluconate, and the lines originating from CaCO_3_. With such a high amount of calcium ions, the diffraction lines should have higher intensities.

The comparative analysis results for *Molekin Osteo* presented in Table 6 confirm a very good accordance of the *2*θ angle values read from the diffraction pattern with the data from the ICDD database. The determined Δ*2*θ values are smaller than 0.2°. It proves the authenticity of the *Molekin Osteo* preparation. *Calcium 500D* should be subject to further studies using other methods.

## Conclusions

The X-ray diffractometric studies carried out for 22 commonly available dietary supplements containing calcium and magnesium allowed for ascertaining that the majority of the analyzed products contain appropriate calcium and magnesium compounds declared by the manufacturers in their specifications. Comparison of *2*θ angle values at which the diffraction lines originating from calcium and magnesium compounds included into compositions of the tested dietary supplements were recorded, with the data from the ICDD database, showed a very good accordance. It indicates use of substances with the same structural parameters or substances being the same crystalline varieties. In most cases, the difference of *2*θ angle values between the experimental data and those from the database is smaller than 0.2°. Abnormalities were found for two dietary supplements containing magnesium ions: *Magnesium KFD Nutrition* and *7 Nutrition Bomb Pre-workout*.

For *Magnesium KFD Nutrition*, the sole visible diffraction line is shifted by more than 0.2° (Δ*2*θ = 0.3628), while for *7 Nutrition Bomb Pre-workout*, no lines of the magnesium compound declared by the manufacturer (magnesium citrate) were identified.

In such a case, when the result of diffractometric analysis is ambiguous or raises some suspicions regarding the authenticity of the product, the tests should be repeated or the analysis expanded with other methods, e.g., IR, UV-Vis, chromatographic methods.

PXRD (Powder X-ray Diffraction) is a fundamental and the most frequently used technique for examination of medical preparations and plays an important role at all stages of pharmaceutical research and development. However, the best practice should consist in an approach in which the XRD studies will be combined with such methods as gas chromatography-mass spectrometry (GC-MS), high performance liquid chromatography (HPLC), Fourier transform infrared spectroscopy (FTIR), or synchrotron radiation (Rendle, 2019).

## Data Availability Statement

All datasets generated for this study are included in the article/supplementary material.

## Author Contributions

IJ was originator of the topic of this manuscript, who prepared all samples to X-ray analysis, who done these measurements and then worked out the results, and wrote the manuscript.

## Conflict of Interest

The author declares that the research was conducted in the absence of any commercial or financial relationships that could be construed as a potential conflict of interest.

## Footnotes

^1^https://serc.carleton.edu/research_education/geochemsheets/techniques/XRD.html (accessed September 06, 2020).

## Appendix

**Table A1:** List of used ICDD PDF2 (Release 2008) cards.

** No.**	**Chemical formula**	**Chemical compound**	**No. PDF card**
1.	MgO	Magnesium oxide	00–001– 235
2.	MgCO_3_	Magnesium carbonate	01–070–8513 01–070–8515
3.	Mg_3_(C_6_H_5_O_7_)_2_	Magnesium citrate	00–001– 0186
4.	C_6_H_10_MgO_6_3H_2_O	Magnesium lactate	00–001–0061
5.	CaCO_3_	Calcium carbonate	00–001–0837 00–001–0628
6.	C_6_H_10_CaO_6_ × 5H_2_O	Calcium lactate	00–005–0101
7.	C_12_H_22_CaO_14_	Calcium gluconate	00–010–0774
8.	NaHCO_3_	Sodium Carbon Hydrogen Oxide	00–001–0909
9.	C_6_H_8_O_6_	Ascorbic acid	00–004–0308
10.	C_6_H_8_O_7_	Citric acid	00–001–0251
11.	(C_6_H_10_O_5_)_x_	Starch	00–030–1912
12.	KCl	Potassium chloride	00–004–1476
13.	C_36_H_70_MgO_4_	Magnesium stearate	00–054–1973
14.	C_3_H_7_NO_2_	Alanine	00–27–1501
